# Supplementary material for: Human herpesvirus 7 integrates into host telomeres via its telomeric repeat arrays
Source: PLoS Pathog. 2026 May 6;22(5):e1014189. doi: 10.1371/journal.ppat.1014189 (PMC13170970; doi:10.1371/journal.ppat.1014189)
Supplement: Attachment — Submitted filename: Response to Reviewers_final.pdf [file ppat.1014189.s008.pdf]

Response to the reviewers

We would like to thank the reviewers for the evaluation of our manuscript. We have addressed their concerns and you can find our point-to-point responses to the reviewers' comments below.

Summary

Reviewer #1:

Cheng et al. demonstrated integration of human herpesvirus 7 (HHV-7) genome into chromosome in CD4 expressing U2OS cells by using fluorescence in situ hybridization (FISH) analysis and Nanopore sequencing. The integration pattern is consistent with those of the previously known viruses, such as HHV-6A/B and Marek's disease virus, which also harbor the terminal telomeric repeat. In addition, authors have cloned the HHV-7 genome as a BAC-maintained DNA in yeast and demonstrated modification of genome in yeast, virus reconstitution from the recombinant DNA through refined methods, and the integration of recombinant genome into the chromosome in U2OS cells. Using the recombinant HHV-7 system, authors revealed that the absence of either the perfect telomeric repeat (pTMR) or imperfect telomeric repeat (impTMR) alone has little effect on the integration, but lack of both significantly diminish the integration efficiency.
This study clarified the possibility of the genome integration of HHV-7, a pathogen latent in our body with many unknown aspects. Experimental techniques, especially the establishment of HHV-7 recombinant system, are excellent. Although most of the findings presented in this manuscript are highly predictable considering the similarity of the genome structures with known viruses, the collected dataset and the novel recombinant system are regarded as highly valuable. In terms of novelty, the authors pointed out one unique feature of the genome integration of HHV-7; "loss of the impTMR can be complemented by the pTMR, and vice versa" (line 299), in contrast to those of HHV-6A/B and MDV. By taking one more step in the analysis, their manuscript will be more valuable for the publication.

We thank the reviewer for his thorough review. We appreciate the kind words on the novel aspects of our manuscript. We performed the analysis as suggested by the reviewer and expanded the discussion (Lines 332ff).

Reviewer #2:

Betaherpesviruses are endemic in the general population with three distinct members involved in human infection. Of these, HHV-7 remains the most understudied of its siblings, HCMV and HHV6a & b. As such, a significant amount of virologic investigations have yet to be performed on this pathogen. In this manuscript, the authors seek to determine if HHV-7 can establish an infection resulting in a viral genome being integrated at the telomeric ends of human chromosomes similar to that found with its roseolavirus counterparts. To this end, the investigators have

generated a stable cell line expressing the HHV-7 receptor (CD4) and can observe long term genomic maintenance of HHV-1 in about 10% of the cell population after a month in which the genome copy number to cell number is ~1. FISH analysis confirmed genomic integration of HHV-7 DNA at the ends of chromosomes at regions that contain telomeres, a finding that was anticipated as the viral genome contains perfect and imperfect repeats of telomeric sequences. Isolation of viral integrated cell lines were further validated by genomic sequencing which revealed that the HHV-7 genome was integrated at telomeric ends in which recombination occurred that deleted one copy each of the Pac1 and Pac2 sequences which are needed for circularization of the genome upon infection. In order to determine the requirement of the perfect and imperfect viral telomeric regions for host cell integration the authors generated a BAC recombinant of HHV-7 by exploiting Yeast recombination of linearized virus followed by bacterial recombination protocols to manipulate the viral genome. Mutants lacking either the perfect telomers, the imperfect telomers or both were generated. While the viral telomeric repeats were found to be expendable for lytic replication of the virus, the double deleted telomeric repeat virus failed to exhibit host cell integration whereas the WT, and each of the single mutants were still capable of integration. The authors conclude that either telomeric region can suffice for viral integration yet a deletion of both renders the virus incapable of integration. Overall this is a well written study and the conclusions are supported by the data presented. That being said, the overall findings are not particularly surprising as one would imagine HHV-7 would behave very similar to its closely related counterparts. While the generation of the BAC containing HHV-7 infectious construct is of value to the community, the study is mostly confirmatory to what one anticipates from roseoloviruses. Some experimental suggestions are provided below.

We thank the reviewer for the positive feedback and highlighting the importance of our novel genetic system for HHV-7. We addressed and followed the experimental suggestions provided by the reviewer (see below).

Reviewer #3:

Human herpesvirus 7 (HHV-7) is a member of the Roseolovirus genus in the betaherpesvirus subfamily. Most adults have been infected since early childhood, and harbor the usually benign virus for the remainder of their lives. HHV-7 genome architecture is similar to that of other roseoloviruses, including human herpesviruses 6A and 6B (HHV-6A and (HHV-6B). An interesting shared feature is the presence of DNA sequences at their genomic termini that are similar to sequences present at the termini of chromosomes of many eukaryotes, (GGGTTA)n. These sequences enable the virus genomes to be incorporated into the chromosomes of cells they infect, including germline cells. About 1% of human are born with inherited chromosomally integrated (ici) genomes of HHV-6A or HHV-6B (iciHHV-6A and iciHHV-6B). Mechanisms and outcomes related to these interesting and intriguing activities are

being investigation in several laboratories.
In this paper, Cheng and colleagues conducted a series of experiments that clearly demonstrate that HHV-7 integrates into human chromosomes via its telomeric repeat arrays.
This is important work. It includes a technically sophisticated and well-executed experimental design. I don’t have a lot of comments on the design, execution, and illustration of the experiments. Unfortunately, the text needs a lot of work. I am providing a detailed list of suggested edits for pages 1-4. It is likely that when these items are repaired, other issues will become visible in the same area, so the authors need to look beyond the current comments prior to resubmitting. While I did not take the time to document everything, the high density of text issues extends through the rest of the text.

We warmly thank the reviewer for highlighting the quality and importance of our work. We really appreciate the editorial suggestion. We applied the suggested edits of the reviewer (page 1-4) and substantially improved the entire text of the manuscript.

Major Issues: Key Experiments Required for Acceptance

Reviewer #1:

1. According to the Nanopore sequencing data in Fig 2, the typical integration pattern of HHV-7 genome uses both impTMR and pTMR keeping the both of DRL and DRR, as illustrated in Fig 2C. However, the recombinant HHV-7 genome lacking either impTMR or pTMR alone still can be integrated, as authors demonstrated in Fig 6. In either case, one of the DRL and DRR may be excluded after integration. As this may be an unexpected and unique feature of HHV-7, authors should provide information regarding the integration patterns for these mutant genomes to further confirm that genome integrations occur in similar or unexpected manners in cases of these mutants.

We thank the reviewer for this constructive comment. As suggested, we set to investigate the integration pattern of the mutant viruses. We first had to designed a new set of gRNAs that would facilitate sequencing the integration site including entire DR region and the (deleted) TMR arrays. In the new experiments, we observed that the TMR are absent as expected, however the reads were not long enough to assess the entire integration site. Extensive optimization of the new gRNA/Cas9-enrichment system as well as sequencing of many integration events would be required to obtain a comprehensive dataset on the mutant viruses. This is beyond the scope of this manuscript and will be performed in a future study. We discussed this aspect in the discussion section in the manuscript (Line 337ff).

Reviewer #2:

1) some experimental specifics are in need of more details. For example,

- what was the MOI used to infect the U2OS-CD4 cells?
  HHV-7 (like HHV-6) is mostly cell-associated and very little cell free virus is produced. Therefore, we (and many colleagues in the field) standardize the inoculum by quantifying the number of infected cells in the inoculum. 5×10^5 infected cells were seeded on the U2OS-CD4 monolayer. The inoculum (suspension cells) was subsequently thoroughly removed and the adherent-U2OS cells further analyzed over time. This information is now included in the manuscript (Line 362ff).

- What % were positive?
  About 8% of the U2OS-CD4 cells were GFP positive (2dpi), indicative for the infection with HHV-7. We included this new data in our manuscript (Line 214 and Line 318ff).

- Were the cells productively infected?

We and others showed that HHV-7 can only productively replicate in CD4+ T cells (e.g. SupT1) [1], and does not productively replicate in U2OS-CD4 cells. HHV-7 does not spread to neighboring cells or form plaques on these cells. We included this information in the manuscript (Line 248ff).

- Do they produce cell free virus?
  No. HHV-7 does not replicate in U2OS-CD4 cells and therefore does not produce cell free virus.

- Do herpesvirus DNA replication inhibitors limit DNA amplification?
  Yes. HHV-7 DNA amplification can be limited by some DNA replication inhibitors, but susceptibility is drug-dependent. Direct viral DNA polymerase inhibitors such as foscarnet (phosphonoformic acid) and cidofovir can inhibit HHV-7 replication in vitro, whereas nucleoside analogues that require viral kinase activation (e.g., acyclovir, ganciclovir) only show weak or minimal activity [2, 3].

- Are lytic transcripts (rt-qPCR) or proteins (WB) observable?
  Yes, transient expression of a late protein (HHV-7 U81) can be observed in U2OS-CD4-GFP cells, despite the absence of productive replication (see below Figure 1).

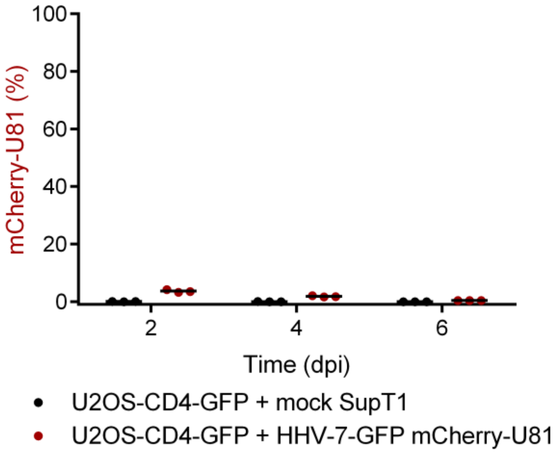

Figure 1: Infection of U2OS-CD4-GFP cells with an HHV-7 mCherry-U81 reporter virus. Mean mCherry-U81 positive percentage with standard deviation are shown for the indicated time points. The data represent three independent experiments.

- Are the low genome numbers seen at 28 days a result of integration or few lytically infected cells?
  Thanks for raising this important point. Our FISH analyses (and U81 expression) show that there is no lytic virus replication at this time point (Fig.1E and 6B) and that the genomes numbers correspond to the integrated virus genomes. We now emphasized this in the manuscript (line 248ff).

- Can the virus be reactivated from the clonal cell lines?

No. The virus cannot be reactivated from the clonal U2OS-CD4 cell lines. For example, we stimulated the cells with TPA and did not see an increase in virus genome copies (see below Figure). We included this information in the manuscript (Line 257ff).

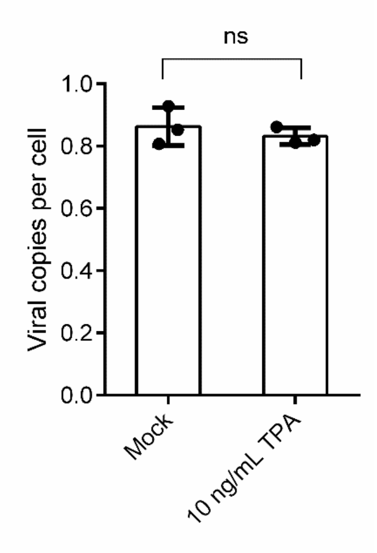

2) it was not clear if the integration sites are found in preferential host chromosomes or just randomly integrated in the telomers of any chromosome.

Thanks for this intriguing comment. Based on our FISH analysis, integration occurred in a wide variety of chromosomes (large, medium and small), suggesting a rather random integration. We are currently developing a high throughput nanopore sequencing approach that will hopefully allow us to sequence hundreds of the integration events, needed to address if integration is truly random or if there is any bias. We discussed this important aspect in the manuscript (Line 326ff).

3) the discussion is just a rehash of the results section and not a comprehensive evaluation of the conclusions that one can derive from the findings.

We thank the reviewer for this comment. The expanded the discussion as suggested by the reviewer (Line 248ff).

Reviewer #3:
As noted above, the paper needs significant re-writing to be able to judge its technical merits. To the extent that I could understand the writing, with good re-writing, the technical aspect of the paper will likely stand as Very Good or Excellent.

We really appreciate the reviewer’s comment and suggestions. The manuscript has been thoroughly revised as suggested by the reviewer.

Minor Issues: Editorial and Data Presentation Modifications

Reviewer #1:

1. Fig. 1D and Lines 85-86, the copy numbers of 74-2 and 74-18 seem to be less than one. Is there any possibility, e.g. one half? Alternatively, does the data indicate the unstable integration of HHV-7 genome?

We thank the reviewer for this keen observation. In our FISH experiment, (almost) all cells had clear staining for the virus genome (Fig. 1E), suggesting that the virus genome is present in every cells. In addition, we observed that the copy number of the virus genome remains stable over time (Fig. 1C). Thanks to your comment, we re-assessed the qPCR standard curves of the viral (U38) and cellular gene (b2M) and noticed that b2M is more efficiently amplified (U38 slope = -2.997, b2M = -3.257), which likely accounts for slightly lower virus genome copies per cell. This aspect is now discussed in the manuscript (Line 106ff, and Line 262).

2. Fig. 2B, the loss of pac2 sequence is difficult to understand from this figure. Readers don't know whether the label "pac2 sequence" is given for the sequence above it or the sequences below it. It could be confirmed after I zoomed in and read the reference sequence. Additional labels are helpful to indicate pTMR before the pac2 sequence, and to indicate pTMR and Telomere for actual sequences e.g. Sample 22-14.

We thank the reviewer for this constructive comment. We have optimized Fig. 2B to provide a better visualization, as well as its figure legend (Line 130).

3. I wonder whether the recombinant HHV-7 genomes can restore the linear genome structures as written in Fig 5A. The BAC DNA becomes circular form by connecting the ends of genome DNA i.e. pac1 and pac2, and it must be properly processed during reconstitution and lytic replication processes. Since the modification sites of ΔpTMR, ΔimpTMR, and ΔTMR are near the end of genomes, it may affect on the concatemer processing. Is there any data or information by which you can confirm the genome structure of each recombinant genome after replication?

We thank the reviewer for this comment. To address the reviewer's comment, we performed Sanger sequencing and could demonstrate that the entire region (incl. pac1, pac2, and TMR modification) are present in the virus genome as expected. In addition, the recombinant viruses replicated comparable to wild type (Fig. 5C), confirming the efficient cleavage and packaging of the recombinant virus genomes. We included this new data in our manuscript (Line 196).

Reviewer #2: (No Response)

Reviewer #3: The number and nature of text modifications needed takes this well beyond “minor”.

The text has been revised as suggested by the reviewer.

L30-31. Start the sentence with the “Using fluorescence …” clause. Delete “Here”.
Done
L 32. “live” to “life”
Done
L32 and L35. Only need to say it is the first such system once.
Updated
L38-39. “90% in humans.”
Updated
L 40. Mention that HHV-7-linked roseola is less frequent than for HHV-6B.
Done
L43. “allow” to “allows”
Updated
L 44. “all herpesviruses.”
Updated
L45-45. “their dsDNA genome”
Updated
L 46. “some herpesviruses, e.g., Epstein Barr virus …”
Updated
L49-50. This is written as though HHV-6 is a single virus.
Updated
L 51. “Marek’s disease virus of chickens”
Updated
L 51. You might reverse the order of MeAHV1 and GaHV3 (put the gallid viruses next to each other).
Updated
L 51-52. “Chromosomal integration ensures that …”
Updated
L53-54. Not quite. “The mechanism used by HHV-7 to maintain its genome during latency is not known.
Updated
L 56-60. Confusing as written. Do you mean that both DRR and DRL contain two perfect TMRs, or one perfect TMR is present in both DRs? How about “both DRs contain a perfect TMR array at their right ends and an imperfect TMR (impTMR) array at their left end. impTMRs are interspersed with related but non-identical hexamers.” Are all impTMRs hexamers?
Updated
L60. “In addition, the DR” to “In addition, DR”
Updated
L61. “sequences required for cleavage …”
Updated

L62. “HHV-6A and HHV-6B”
Updated
L64. Change “the role … remain” to either “the roles of … remain” or "the role … remains”.
Updated

L65-72 is one of the most important paragraphs in the paper. It is difficult to understand as written. Line 65 implies that latency is established in infected cells prior to chromosomal integration of the virus genome (“can integrate into the telomeres of latently infected cells”, L65-66). This wording implies that telomeric integration is not part of the mechanism for establishing latency. If that is not what you meant, how about some reordering:
“To assess the role of the TMR in the virus life cycle, we generated the first reverse genetics system for HHV-7 using TAR cloning in *Saccharomyces cerevisiae*. This system facilitated generation of pTMR and impTMR mutants that enabled investigation of their roles in virus replication. We found that both TMR are completely dispensable for virus replication but are crucial for integration of the virus genome into host telomeres.”
We thank the reviewer for raising this important point. We have updated this paragraph.

L71-72. I am not sure how “while pTMR and impTMR can complement each other in this process” fits in. “Can” implies that they don’t always complement each other, which suggests the possibility of numerous unmentioned alternatives.
Suggestion for L72-74: “Our study provides important insights into HHV-7 biology as well as a genetic system that will help advance understanding of the complex biology of this ubiquitous human pathogen that has learned enough to co-exist relatively peacefully with most humans, most of the time.”
We thank the reviewer for this comment. We have updated this paragraph.

References:
1. Miyake F, Yoshikawa T, Sun H, Kakimi A, Ohashi M, Akimoto S, et al. Latent infection of human herpesvirus 7 in CD4+ T lymphocytes. Journal of Medical Virology. 2006;78(1):112-6. doi: 10.1002/jmv.20511.
2. Zhang Y, Schols D, De Clercq E. Selective activity of various antiviral compounds against HHV-7 infection. Antiviral Res. 1999;43(1):23-35. doi: 10.1016/s0166-3542(99)00031-5. PubMed PMID: 10480261.
3. De Clercq E, Naesens L, De Bolle L, Schols D, Zhang Y, Neyts J. Antiviral agents active against human herpesviruses HHV-6, HHV-7 and HHV-8. Rev Med Virol. 2001;11(6):381-95. doi: 10.1002/rmv.336. PubMed PMID: 11747000.
