# Supplementary material for: Human herpesvirus 7 integrates into host telomeres via its telomeric repeat arrays
Source: PLoS Pathog. 2026 May 6;22(5):e1014189. doi: 10.1371/journal.ppat.1014189 (PMC13170970; doi:10.1371/journal.ppat.1014189)
Supplement: Attachment — Submitted filename: 260303 review PlosPathogens Cheng et al.pdf [file ppat.1014189.s007.pdf]

Summary:

Cheng and colleagues provide convincing evidence that human herpesvirus 7 (HHV-7) can integrate into host telomeres in persistently infected cells, combining fluorescence in situ hybridization with long-read nanopore sequencing. A major strength of this work is the establishment of the first HHV-7 reverse genetics platform, which will be broadly useful to the field. Using this system, the authors further dissect the contribution of the perfect and imperfect telomeric repeat arrays, showing that these elements are dispensable for lytic replication but are critical for efficient integration and for long-term genome maintenance during persistent infection. Although several aspects of the biology are conceptually consistent with what is known for other roseoloviruses, the datasets are solid and the technical advances and resources provided here represent a clear contribution to the community. Importantly, the authors have also responded appropriately to the comments raised by the three reviewers and improved the manuscript accordingly. Overall, the conclusions are supported by the data, and I recommend acceptance after minor revision to further improve clarity of presentation and the framing of novelty (particularly in comparison with HHV-6A, HHV-6B, and other telomere-associated herpesviruses), and to address a small number of remaining points that can be handled with limited additional work.

Major Issues:

1. The study shows HHV-7 telomeric integration in a cell-line model, but the manuscript does not adequately address how often HHV-7 integration is detected in human genomes. In contrast to HHV-6A/6B (for which inherited integration is reported at a measurable global frequency), HHV-7 integration in humans is scarcely documented (the cited Prusty et al., JGV 2017 reports only two cases). If population-level estimates or screening data exist, please cite and summarize them in the Introduction/Discussion. If not, explicitly state this limitation and discuss plausible reasons why HHV-7 integration is not maintained or detected in humans at HHV-6A/6B-like frequencies.
2. Figure 6B provides key evidence for integration, but the frequency of the shown FISH pattern is not quantified for each mutant. Please report the percentage of cells displaying the integration-associated pattern for each genotype. In addition, compare the HHV-7 integration efficiency with that reported for HHV-6A/6B in comparable U2OS-based systems (e.g., ~5–20% in Gravel et al., JVI 2017) and discuss possible reasons for any differences.

Minor Issues:

1. **(Fig. 6C):** The presentation/legend needs clarification. The plot appears boxplot-like, yet the legend states that "mean HHV-7 genome copies … of 3 independent experiments" are shown. Please revise the legend (and figure if needed) to explicitly define what the center line and box/whiskers represent (mean vs median; SD/SEM vs range/IQR), and indicate n=3. Ideally, show the individual data points for the three experiments.
