# Supplementary material for: Human herpesvirus 7 integrates into host telomeres via its telomeric repeat arrays
Source: PLoS Pathog. 2026 May 6;22(5):e1014189. doi: 10.1371/journal.ppat.1014189 (PMC13170970; doi:10.1371/journal.ppat.1014189)
Supplement: Attachment — Submitted filename: Rebuttal letter_Response to Reviewers_final 2.pdf [file ppat.1014189.s009.pdf]

Response to the reviewers

We would like to thank the reviewers for the evaluation of our manuscript. We have addressed the concerns of the reviewers and you can find our point-to-point responses to the reviewers' comments below.

Reviewer #1:

Cheng and colleagues provide convincing evidence that human herpesvirus 7 (HHV-7) can integrate into host telomeres in persistently infected cells, combining fluorescence in situ hybridization with long-read nanopore sequencing. A major strength of this work is the establishment of the first HHV-7 reverse genetics platform, which will be broadly useful to the field. Using this system, the authors further dissect the contribution of the perfect and imperfect telomeric repeat arrays, showing that these elements are dispensable for lytic replication but are critical for efficient integration and for long-term genome maintenance during persistent infection. Although several aspects of the biology are conceptually consistent with what is known for other roseoloviruses, the datasets are solid and the technical advances and resources provided here represent a clear contribution to the community. Importantly, the authors have also responded appropriately to the comments raised by the three reviewers and improved the manuscript accordingly. Overall, the conclusions are supported by the data, and I recommend acceptance after minor revision to further improve clarity of presentation and the framing of novelty (particularly in comparison with HHV-6A, HHV-6B, and other telomere-associated herpesviruses), and to address a small number of remaining points that can be handled with limited additional work.

We warmly thank the reviewers for highlighting the significant potential and importance of our work. We have addressed their concerns and updated in the manuscript accordingly.

Major Issues:

1. The study shows HHV-7 telomeric integration in a cell-line model, but the manuscript does not adequately address how often HHV-7 integration is detected in human genomes. In contrast to HHV-6A/6B (for which inherited integration is reported at a measurable global frequency), HHV-7 integration in humans is scarcely documented (the cited Prusty et al., JGV 2017 reports only two cases). If population-level estimates or screening data exist, please cite and summarize them in the Introduction/Discussion. If not, explicitly state this limitation and discuss plausible reasons why HHV-7 integration is not maintained or detected in humans at HHV-6A/6B-like frequencies.

Thank you for raising this intriguing aspect. As mentioned by the reviewer, HHV-7 integration is scarcely documented with only the case(s) published by Prusty et al. To our knowledge, none of the large-cohort/population studies observed endogenous HHV-7 in their cohorts. This rare incidence could be due to the inability of the virus to infect germ cells or that cells do not survive HHV-7 infection. This also suggests that HHV-7 uses its integration mechanism predominantly for latency but not endogenization, as is typical for most other herpesviruses with telomeric repeat arrays at their genome ends [1-3]. We discussed this intriguing point in our manuscript (Line 278ff).

2. Figure 6B provides key evidence for integration, but the frequency of the shown FISH pattern is not quantified for each mutant. Please report the percentage of cells displaying the integration-associated pattern for each genotype. In addition, compare the HHV-7 integration efficiency with that reported for HHV-6A/6B in comparable U2OS-based systems (e.g., ~5–20% in Gravel et al., JVI 2017) and discuss possible reasons for any differences.

We thank the reviewer for pointing this out. As suggested by the reviewer, we quantified the integration frequency of the mutant viruses. FISH analyses (n=3) revealed that integration of the ΔpTMR and ΔTMR were significantly impaired. We included this new data below and, in the manuscript (Figure 6D and 6E, Line 223ff and 237ff).

The integration frequency observed in our HHV-7 system is about 2-3 %, which is indeed slightly lower than the 5-20 % previously reported for HHV-6A/B (Gravel et al., JVI 2017). The lower integration frequency could be caused by several factors, including differences i) in the integration efficiency, ii) the viral genome (e.g. U94 is encoded by HHV-6A/B but not HHV-7), and iii) expression latency gene that could affect cell proliferation. We now discuss this aspect in the manuscript (Line 273ff).

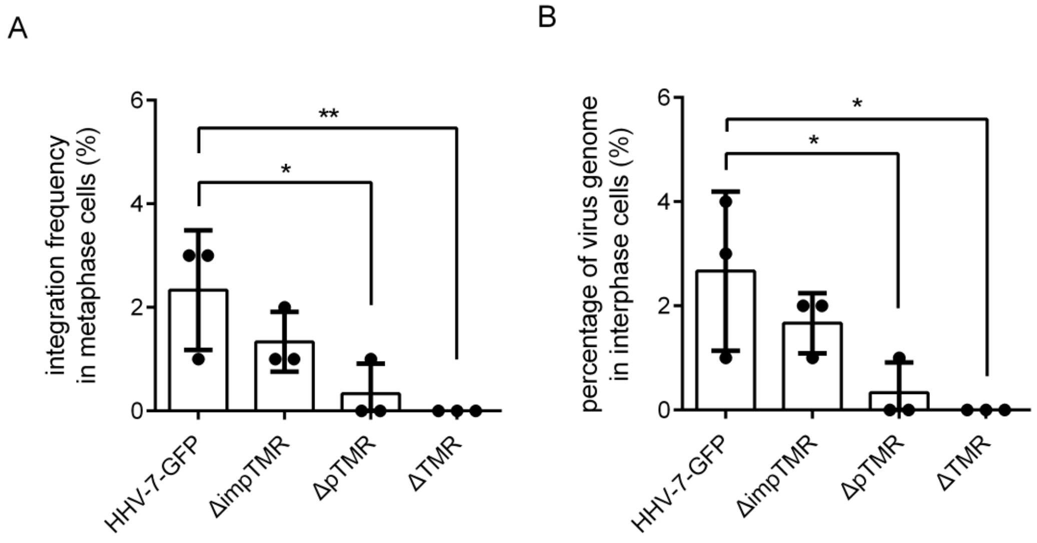

Fig. 1: A. The integration frequency was determined by analyzing at least 100 metaphases per sample. Asterisks (*) indicate significant differences between HHV-7-GFFP and ΔpTMR, as well as HHV-7-GFP and ΔTMR groups ($p < 0.05$, Dunnett's multiple comparisons test). Data are presented as the means of three independent experiments with standard errors. B. 100 interphase nuclei were examined for the presence of HHV-7. Asterisks indicate significant differences between HHV-7-GFFP and ΔpTMR, as well as HHV-7-GFP and ΔTMR groups ($p < 0.05$, Dunnett's multiple comparisons test). Data are presented as the means of three independent experiments with standard errors.

Minor Issues:

1. **(Fig. 6C):** The presentation/legend needs clarification. The plot appears boxplot-like, yet the legend states that “mean HHV-7 genome copies … of 3 independent experiments” are shown. Please revise the legend (and figure if needed) to explicitly define what the center line and box/whiskers represent (mean vs median; SD/SEM vs range/IQR), and indicate n=3. Ideally, show the individual data points for the three experiments.

We thank the reviewer for this constructive comment. We have optimized Fig. 6C to provide a better visualization, as well as its figure legend (Line 237ff).

Reviewer #2:

The authors have sufficiently addressed any concerns raised in initial review. The modified manuscript is sufficiently improved and no further issues remain.

We thank the reviewers for the positive feedback and taking the time to review our manuscript.

References:

1. You Y, Kheimar AM, Vychodil T, Kossak L, Sabsabi MA, Conradie AM, et al. Telomeric repeats in the commercial SB-1 vaccine facilitate viral integration and contribute to vaccine efficacy. NPJ Vaccines. 2024;9(1):154. Epub 2024/08/22. doi: 10.1038/s41541-024-00945-6. PubMed PMID: 39169010; PubMed Central PMCID: PMCPMC11339279 commercial or financial relationships that could be construed as a potential conflict of interest.

2. Denesvre C, You Y, Remy S, Vychodil T, Courvoisier K, Penzes Z, et al. Impact of viral telomeric repeat sequences on herpesvirus vector vaccine integration and persistence. PLoS Pathog. 2024;20(5):e1012261. Epub 2024/05/28. doi: 10.1371/journal.ppat.1012261. PubMed PMID: 38805555; PubMed Central PMCID: PMCPMC11161090 other authors declare that they have no conflict of interest.

3. You Y, Vychodil T, Aimola G, Previdelli RL, Gobel TW, Bertzbach LD, et al. A Cell Culture System to Investigate Marek's Disease Virus Integration into Host Chromosomes. Microorganisms. 2021;9(12). Epub 2021/12/25. doi: 10.3390/microorganisms9122489. PubMed PMID: 34946091; PubMed Central PMCID: PMCPMC8706938.
