# Supplementary material for: Human herpesvirus 7 integrates into host telomeres via its telomeric repeat arrays
Source: PLoS Pathog. 2026 May 6;22(5):e1014189. doi: 10.1371/journal.ppat.1014189 (PMC13170970; doi:10.1371/journal.ppat.1014189)
Supplement: Attachment — Submitted filename: PPATHOGENS-D-25-02654_RevComments.docx [file ppat.1014189.s005.docx]

Comments and suggestions, in order of occurrence, from page 1 to the top of page 4 :

L30-31.  Start the sentence with the “Using fluorescence …” clause.  Delete “Here”.

L 32.  “live” to “life”

L32 and L35.  Only need to say it is the first such system once.

L38-39.  “90% in humans.”

L 40.  Mention that HHV-7-linked roseola is less frequent than for HHV-6B.

L43.  “allow” to “allows”

L 44.  “all herpesviruses.”

L45-45.  “their dsDNA genome”

L 46.  “some herpesviruses, e.g., Epstein Barr virus …”

L49-50.  This is written as though HHV-6 is a single virus.

L 51.  “Marek’s disease virus of chickens”

L 51.  You might reverse the order of MeAHV1 and GaHV3 (put the gallid viruses next to each other).

L 51-52.  “Chromosomal integration ensures that …”

L53-54. Not quite.  “The mechanism used by HHV-7 to maintain its genome during latency is not known.

L 56-60.  Confusing as written.  Do you mean that both DRR and DRL contain two perfect TMRs, or one perfect TMR is present in both DRs?  How about “both DRs contain a perfect TMR array at their right ends and an imperfect TMR (impTMR) array at their left end.  impTMRs are interspersed with related but non-identical hexamers.”  Are all impTMRs hexamers?

L60.  “In addition, the DR” to “In addition, DR”

L61.  “sequences required for cleavage …”

L62.  “HHV-6A and HHV-6B”

L64.  Change “the role … remain” to either “the roles of … remain” or "the role … remains”.

L65-72 is one of the most important paragraphs in the paper.  It is difficult to understand as written.  Line 65 implies that latency is established in infected cells prior to chromosomal integration of the virus genome (“can integrate into the telomeres of latently infected cells”, L65-66).  This wording implies that telomeric integration is not part of the mechanism for establishing latency.  If that is not what you meant, how about some reordering:

“To assess the role of the TMR in the virus life cycle, we generated the first reverse genetics system for HHV-7 using TAR cloning in *Saccharomyces cerevisiae*. This system facilitated generation of pTMR and impTMR mutants that enabled investigation of their roles in virus replication.  We found that both TMR are completely dispensable for virus replication but are crucial for integration of the virus genome into host telomeres.”

L71-72.  I am not sure how “while pTMR and impTMR can complement each other in this process” fits in. “Can” implies that they don’t always complement each other, which suggests the possibility of  numerous unmentioned alternatives.

Suggestion for L72-74:  “Our study provides important insights into HHV-7 biology as well as a genetic system that will help advance understanding of the complex biology of this ubiquitous human pathogen that has learned enough to co-exist relatively peacefully with most humans, most of the time.”
